# Combining Artificial Neural Network and Ordinary Kriging to Predict Wetland Soil Organic Carbon Concentration in China’s Liao River Basin

**DOI:** 10.3390/s20247005

**Published:** 2020-12-08

**Authors:** Yingdong Kang, Xiaoyan Li, Dehua Mao, Zongming Wang, Mingxuan Liang

**Affiliations:** 1 College of Earth Science, Jilin University, Changchun 130100, China; kangyd18@mails.jlu.edu.cn (Y.K.); lxyan@jlu.edu.cn (X.L.); 2 Key Laboratory of Wetland Ecology and Environment, Northeast Institute of Geography and Agroecology, Chinese Academy of Sciences, Changchun 130102, China; zongmingwang@iga.ac.cn (Z.W.); Liangmx0911@163.com (M.L.); 3 National Earth System Science Data Center, Beijing 100101, China; 4 School of Geography and Environment, Jiangxi Normal University, Nanchang 330022, China

**Keywords:** soil organic carbon concentration, wetland, digital soil mapping, artificial neural network, remote sensing

## Abstract

Accurate prediction of wetland soil organic carbon concentration and an understanding of its controlling factors are important for studying regional climate change and wetland carbon cycles; with that knowledge mechanisms can be put in place that are conducive to sustainable ecosystem management for environmental health. In this study, a hybrid approach combining an artificial neural network and ordinary kriging and 103 soil samples at three soil depth ranges (0–30, 30–60, and 60–100 cm) were used to predict wetland soil organic carbon concentration in China’s Liao River Basin. The model evaluation indicated that a combination of artificial neural network and ordinary kriging and limited soil samples achieved good performance in predicting wetland soil organic carbon concentration. Wetland soil organic carbon concentration in the Liao River Basin has apparent spatial and vertical heterogeneities with values decreasing from southeast to northwest and concentrates present mainly in the topsoil (0–30 cm). Mean wetland soil organic carbon concentration values at the three soil depths were 10.43 ± 0.38, 7.93 ± 0.25, and 7.61 ± 0.22 g/kg, respectively, which are smaller than those over other wetland regions in Northeast China. Terrain aspect contributed the most in predicting wetland soil organic carbon concentration at each of the three soil depths, followed by normalized difference vegetation index at 0–30 cm and mean annual precipitation at 30–60 and 60–100 cm. This study provides a framework method and baseline to quantify the soil organic carbon concentration dynamics in response to climatic and anthropogenic drivers.

## 1. Introduction

In the context of global warming and environmental degradation, carbon sequestration and emissions from wetlands have caused widespread concerns of governments and academics worldwide [1]. Accurate prediction of wetland soil organic carbon concentration (SOCc) and investigation of its influence factors are important for an understanding of the regional wetland carbon cycling and climate change; with that knowledge mechanisms can be put in place that are conducive to sustainable ecosystem management for environmental health [2,3].

The accuracy of wetland SOCc prediction is mainly restricted by the forecast methods and input data availability [4]. Digital soil mapping (DSM) was proposed to predict the spatial and vertical variations of wetland SOCc in regional areas due to the low cost by reducing sampling data sets and environmental factors [5,6]. In the past decades, various machine learning (ML) methods, including support vector regression (SVR) [7], boosted regression tree [8], random forest (RF) [9], and artificial neural networks (ANNs) [10,11], were applied to spatially predict soil parameters at different scales. The application of ANNs could be one of the most effective methods because of its strong performance in characterizing the nonlinear relationships between soil parameters and environmental variables [12,13]. Multisource geospatial data correlated with SOCc, including topographic [14], meteorological [15], and remotely sensed data [16], can be combined to predict SOCc and reveal the relationship between SOCc and environmental variables. For example, Emadi et al. reported that a deep neural network method had an excellent performance in predicting soil organic carbon in northern Iran by inputting 105 environmental variables as predictors [5]. Wang et al. compared different ML algorithms to estimate soil organic carbon stocks in the semi-arid rangelands of eastern Australia [8]. By integrating climatic, terrain, and remote sensing data, the performance of ML methods to predict SOCc was significantly improved [12]. In most cases, ML methods employing diverse environmental variables can obtain more accurate predictions than traditional linear and geostatistical methods (i.e., inverse distance weighting, kriging), especially at a broad scale with limited samples because of their strong performance in gaining more information from the nonlinear relationships between SOCc and the environmental variables.

Soil, especially SOCc, varies in different places with high complexity [17]. Therefore, these ML methods have a critical disadvantage—SOCc prediction considers only the input environmental variables at corresponding locations but neglects the spatial autocorrelation among the soil samples. The principle of ordinary kriging (OK) depends on such spatial autocorrelation [18]. The spatial autocorrelation of measured values can be incorporated by integrating an OK method that interpolates the residuals from an ANN. Therefore, the combination of ANNs and OK methods could achieve high prediction accuracy and low error [19]. Although many algorithms and environmental variables have been developed to predict soil properties, the development of techniques that comprehensively consider environmental factors and spatial autocorrelation is necessary to enhance the quality of thematic soil maps. However, a method that combines ANN and OK methods using multisource geospatial data to predict wetland SOCc is still absent and is seldom reported in the literature [19].

China’s Liao River Basin (LRB) is an important region in Northeast Asia because of the widely distributed coastal and inland wetlands and the prominent responses of the regional carbon cycle to climatic changes and human activities [20]. These realities create demand for studying the current wetland SOCc distribution and their interaction with various environmental factors for this region. However, previous studies mainly paid attention to surface soil organic carbon of wetlands over the Liao Estuary, which did not comprehensively investigate the spatial and vertical variance and the influence factors of wetland SOCc at the regional level [21].

For an accurate spatial prediction of wetland SOCc, in this study, a hybrid approach combining ANN and OK (ANN-OK), by employing multisource geospatial data and measured soil data, was used, and the spatial and vertical patterns of wetland SOCc were examined in the LRB. The purposes of this study are to (1) test the performance of ANN-OK in predicting wetland SOCc; (2) reveal spatial and vertical patterns of wetland SOCc in the LRB; and (3) examine the impacts of various environmental factors on wetland SOCc. The hybrid method and employed environmental variables are expected to provide a framework for the prediction of soil parameters in other regions and a baseline to evaluate the soil carbon changes in response to climatic and anthropogenic drivers in the future.

Given these objectives, specifically, the spatial prediction of wetland SOCc was firstly achieved with ANN. Furthermore, the residual error calculated by samples were then spatially interpolated to improve the accuracy of the ANN achieved prediction. We also compared the performance of different algorithms (OK, ANN, and ANN-OK) to document the applicability of the ANN-OK. Based on the ANN-OK predictions, the spatial and vertical distributions of wetland SOCc were mapped and relative importance analysis of environmental factors was conducted. Finally, the advantages of the prediction model, spatial heterogeneity of wetland SOCc in LRB, and the influence of environmental factors were discussed. 

## 2. Materials and Methods

### 2.1. Study Area

**Figure 1:**
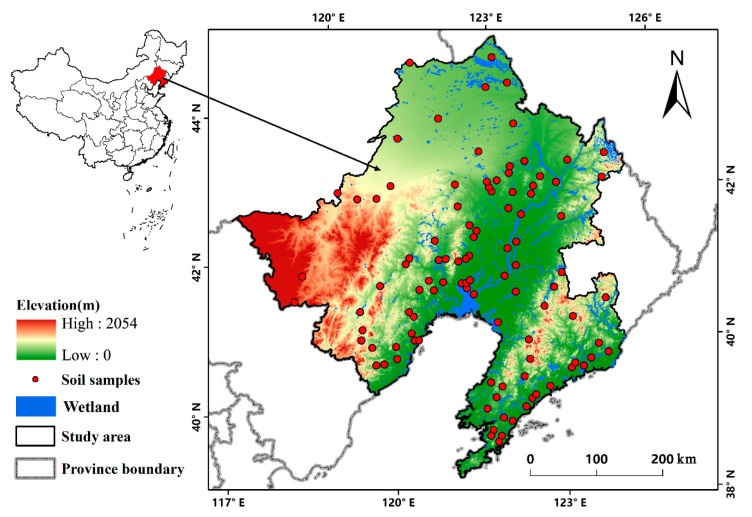
Location of the Liao River Basin (LRB) in China and the distribution of wetland and soil samples in the study area.

The LRB (117°47′–125°06′ E, 38°43′–44°30′ N) in southern Northeast China, with a total area of approximately 187,000 km^2^, is an important part of the Northeast Plains (Figure 1). The study area is characterized by a semi-humid temperate monsoon climate with a mean annual temperature (MAT) of 7–13 °C from north to south, a mean annual precipitation (MAP) of 352–954 mm and a mean annual relative humidity (MAH) of 36–82% from northwest to southeast. This basin is one of the most important wetland distribution regions in China, where many migratory waterbirds live and breed in the East Asian-Australasian Flyway [22]. Wetlands, which refer only to the vegetated wetland in this study, are mainly concentrated at the lower reaches of the Liao River, especially in the Liao Estuary National Nature Reserve [23]. Dominant species are Reed (*Phragmites australis*) and Suaeda (*Suaeda glauca*).

### 2.2. Soil Sampling and Determination

Before the field survey, preliminary sampling sites were designed based on the wetland distribution in the LRB. Due to the road accessibility, 103 soil samples were obtained from September to October 2017 (Figure 1). Each sample consisted of three soil repeats collected by standard containers, and each repeat included 3 soil depths: 0–30, 30–60, and 60–100 cm [16]. The coordinates of every soil sample were recorded by the global positioning system (GPS). Wetland SOCc for each soil sample at different depths was represented by the average values of these three corresponding soil sample repeats. All soil samples were air-dried and sieved to pass a 2 mm mesh to determine the SOCc by the potassium dichromate external heating oxidation method. In the procedure, each soil sample was heated with potassium dichromate at approximately 100–105 °C to obtain carbon dioxide by oxidizing the organic matter [24]. Then, dichromate ions were reduced. The SOCc can be calculated by various dichromate ion numbers in the carbon oxidation process.

### 2.3. Wetland Distribution Dataset

The wetland distribution dataset was obtained from the wetland ecology and environment data center of the Chinese Academy of Sciences (www.igadc.cn). This dataset was generated by an object-oriented classification method and the eCognition Developer software. There are four key steps in the process of classification: multiresolution segmentation, decision rule-based classification, preliminary result revision, and product accuracy evaluation [25]. The accuracy of the primary wetland distribution results revised by visual interpretation and ground survey samples was assessed to be 94%.

### 2.4. Environmental Variables

The variation of wetland SOCc can be affected by soil formation factors, including climate, soil properties, terrain, organisms, and parent materials [16]. In this study, 11 environmental variables (Table 1) extracted from remote sensing, meteorological, and topographical data were combined to predict wetland SOCc.

#### 2.4.1. Remote Sensing Data

The growth status of surface vegetation, which can be detected by the spectral characteristics of remote sensing data, directly affects the difference in input soil organic matter and results in a significant difference in SOCc [26]. In this study, remote sensing data, including Landsat 8 operational land imager (OLI) and Moderate Resolution Imaging Spectroradiometer (MODIS) images, were acquired from the Google Earth Engine platform (https://earthengine.google.com/). Landsat 8 OLI images were acquired at a 30 m spatial resolution and with less than 10% cloud coverage in the summer of 2017 (July to September). Visible-red (B4, 0.64–0.67 μm), near-infrared (B5, 0.85–0.88 μm), and short–wave infrared (B6, 1.57–1.65 μm) bands reflect the vegetation growth, coverage, and biomass, respectively. The average normalized difference vegetation index (NDVI) and average enhanced vegetation index (EVI) from July to September at 1000 m spatial resolution were derived from MODIS vegetation indices 16-Day product in 2017 without cloud cover. All three bands and two spectral indices were calculated as vegetation variables.

#### 2.4.2. Meteorological Data

Climate change affects plant productivity, litter rate, and microbial activity through temperature and precipitation changes, which has an important impact on soil organic carbon accumulation [27]. MAP, MAT, and MAH were first calculated based on the recorded meteorological data during 2008–2017, from the China Meteorological Data Sharing Service System (http://data.cma.cn/). These climatic variables were then spatially interpolated to cover the whole study area at a 1000 m spatial resolution by the inverse distance weighting method (IDW), which is a popular and practical method to obtain a spatial continuous climatic map [28].

#### 2.4.3. Terrain Data

Topographic factors have high potential to greatly explain the change of wetland SOCc in the LRB due to high spatial variability in the study area [29]. The digital elevation model (DEM) with 30 m spatial resolution released by ASTER GDEM V2 (http://glovis.usgs.gov/) was obtained to extract the relevant terrain factors. Three topographic variables were collected: altitude, slope gradient, and terrain aspect.

**Figure 2:**
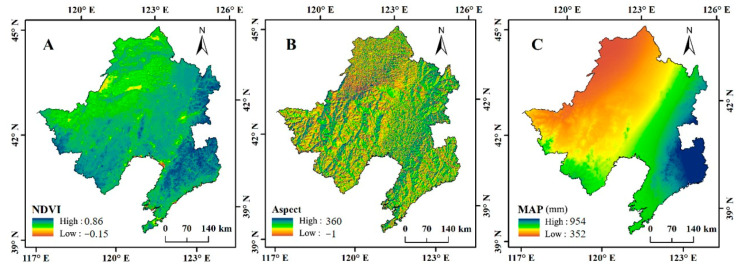
Three examples of environmental variates to predict wetland soil organic carbon concentration (SOCc): (**A**) normalized difference vegetation index (NDVI), (**B**) aspect (the value of −1 means flat), and (**C**) mean annual precipitation (MAP).

All the above related environmental data containing the spatial and attributive values to predict wetland SOCc in the LRB were imported into a GIS database. The Albers equal-area conic WGS84 coordinate system was used to unify these digitized layers from different sources. To match the resolutions of all layers, the bilinear interpolation method was used to resample the spatial resolution of all layers to 1000 m. Moreover, the attributive values of related environmental factor layers were extracted and assigned to all sampling points and used as preliminary indicators for training prediction algorithms. Figure 2 shows the spatial distribution of some relatively important environmental variables related to wetland SOCc, including NDVI, aspect, and MAP.

### 2.5. Selection and Standardization of Optimal Environmental Variables

Too many input environmental variables, which may have good fitting but no predictive ability, are sometimes chance correlations and often represent multicollinearity and overfitting [30]. These problems are well known and important. The relationships between wetland SOCc and environmental variables must be determined before establishing the prediction model of wetland SOCc. When the number of variables is huge relative to the number of samples, variable reduction or selection is commonly used to reduce the data redundancy, multicollinearity, and overfitting [31]. In our study, the Pearson correlation coefficient was used to select the optimal environmental variables based on the rule of value < 0.6 between environmental variables [32]. Furthermore, standardization was performed to eliminate the dimensional effects of input data from different sources and to make the value for each selected environmental variable conform to the standard normal distribution. The zero-mean normalization method was used as the standardization method, which standardizes the original data set into a data set with the mean value of 0 and the variance value of 1 as follows:

(1) $$X_{st}=\;\frac{X-\;\mu }{\sigma }$$

where $X_{st}$ represents the standardized data; X is the original data; and μ and σ are the mean and variance of the original data set, respectively. 

### 2.6. Combination Method to Predict Wetland SOCc

**Figure 3:**
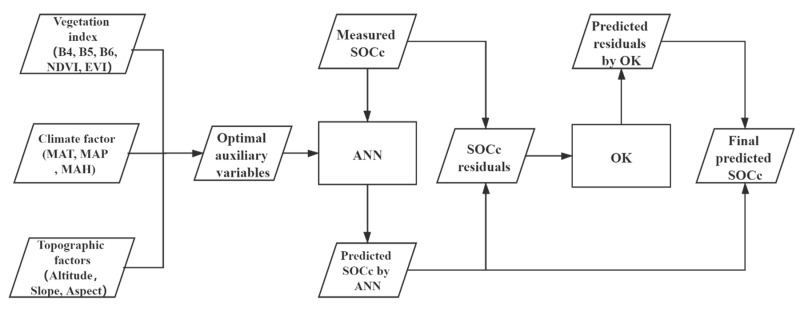
Flow diagram of wetland soil organic carbon concentration (SOCc) prediction by combining an artificial neural network (ANN) and ordinary kriging (OK).

In this study, a hybrid method that combines an ANN and OK was used to predict wetland SOCc (Figure 3). The measured sampling data and optimal environmental variables were first used to predict wetland SOCc in the LRB by an ANN. Then, OK was used to interpolate wetland SOCc residuals calculated by ANN, to the whole spatial extent. The final predicted wetland SOCc was obtained as the sum of wetland SOCc predicted by the ANN and residuals interpolated by the OK.

#### 2.6.1. Spatial Prediction of Wetland SOCc by ANN

The 103 soil samples were randomly assigned to two groups. The training set (n = 76) was used to train the ANN model, and the validation set (n = 27) was used to validate the accuracy of prediction results. A radial basis function (RBF) network, which is a multi-layer feed-forward ANN trained by the error backpropagation algorithm, was selected to predict wetland SOCc, with optimal variables for its simple structure and strong plasticity [33]. Specifically, there are three layers of RBF networks: an input layer, a hidden layer with non-linear RBF activation function, and a linear output layer. In this study, the prediction results of the RBF network are obtained by the following formula:

(2) $$Z_{ANN}=\;\overset{k}{\underset{j=1}{\sum }}\omega_{j}\varphi_{j}\left(Y_{j}\right)+b$$

where $Z_{ANN}$ is the wetland SOCc predicted by the ANN; $Y_{j}$ is a tensor of environmental variables that are calculated from the input layer to the hidden layer; *k* is the number of hidden neurons; $\omega_{j}$ is the connecting weight between an output neuron and a hidden neuron; and *b* is the bias of the model. Both $\omega_{j}$ and *b* are the network parameters, which are iterated by the network’s own calculation; and $\varphi_{j}$ is the Gaussian function, which is selected as an activation function for the hidden neurons and is expressed as follows:

(3) $$\varphi_{j}\left(Y_{j}\right)=\;\exp\left(\frac{-\|Y_{j}-c_{j}{\|}^{2}}{2\sigma^{2}}\right)$$

where $\left\|Y_{j}-c_{j}\right\|$ represents the Euclidean norm between $Y_{j}$ and $c_{j}$; $c_{j}$ is the center of the hidden neuron; and *σ* is the extent of all hidden neurons. The term *σ* can be calculated by:

(4) $$\sigma =\;\frac{d_{max}}{\sqrt{2k}}$$

where $d_{max}$ is the maximum distance between any pair of hidden neurons, and *k* is the number of hidden neurons.

In this RBF network, four types of parameters were iterated by the model calculation to suit the network to a specific task: network bias *b*, output weight $\omega_{j}$, center tensor $c_{j}$, and width parameters *σ*. The number of hidden neurons increased from zero to the most appropriate one that best performed in both training and testing. The network training process is described in detail as follows:

Step 1: Input the environmental variables and simulate the RBF network.

Step 2: Find the input tensor with a random error and add an RBF with weights.

Step 3: Redesign the weights of each neuron and bias using the gradient descent algorithm to minimize the error until the mean squared error reaches the target range.

#### 2.6.2. Residual Interpolation by OK

Considering the spatial autocorrelation, the residuals calculated by the RBF network were used for the stochastic interpolation. The residual only retains the spatial variability decided by the spatial autocorrelation among surrounding measurements, while the complicated connections between the actual wetland SOCc and environmental variables were eliminated as the prediction outcome of the ANN. The residual is defined by

(5) $$r\left(x_{i}\right)=\;\hat{Z}\left(x_{i}\right)-Z_{ANN}\left(x_{i}\right)$$

where *r* is the residual, $\hat{Z}$ is the measured value, and $Z_{ANN}$ is the prediction value calculated by the ANN.

OK is the most common kriging method in practice that has linear optimal proper interpolation with a minimum mean squared error [18]. In this study, OK was used to map the residual calculated by the RBF network. The spatial dependence between sampling sites is usually described using the experimental semi-variogram [34], which can be defined as half of the variance of the difference between the data pairs separated by the lag distance *h* as follows:

(6) $$\gamma \left(h\right)=\;\frac{1}{2N\left(h\right)}\;\overset{N\left(h\right)}{\underset{i=1}{\sum }}{\left[r\left(x_{i}\right)-r\left(x_{i}+h\right)\right]}^{2}$$

where γ(h) is the experimental semi-variance for all pairs within lag distance *h*, and N(h) is the number of sampling site pairs separated by lag distance *h*. This formula is used for the OK interpolation of the ANN residuals.

The final predicted wetland SOCc, *Z*, was obtained as the total of ANN prediction, $Z_{ANN}$, and OK prediction of residuals, $r_{OK}$, as follows:

(7) $$Z\left(x_{i}\right)=\;Z_{ANN}\left(x_{i}\right)+r_{OK}\left(x_{i}\right)$$

To test the approach provided in this study, both OK and ANN were used to make separate predictions, and their prediction accuracies were then compared. In this study, SPSS 24.0 was used to select and standardize the optimal environmental variables, MATLAB 2017a was used to train the ANN, and GS+ 9.0 was used to perform the OK interpolation.

### 2.7. Evaluation of the Accuracy of Prediction Models

To assess the accuracy of the hybrid method, the predictions of wetland SOCc using single ANN and OK were performed. Cross-validation was performed to assess the performances of the three methods. Three indices used in this study include mean error (ME), root mean square error (RMSE), and correlation coefficient (*r*) between the measured and predicted parameters. Both the ME and RMSE represent accuracy, uncertainty, and stability, while *r* denotes the correlation between predicted and measured values [15]. They are computed as follows:

(8) $$ME=\;\frac{1}{n}\;\overset{n}{\underset{i=1}{\sum }}\left[\hat{Z}\left(x_{i}\right)-Z\left(x_{i}\right)\right]$$

(9) $$RMSE=\;\sqrt{\frac{1}{n}\;\sum_{i=1}^{n}{\left[\hat{Z}\left(x_{i}\right)-Z\left(x_{i}\right)\right]}^{2}}$$

(10) $$r=\;\frac{{\left\{\sum_{i=1}^{n}\left[\hat{Z}\left(x_{i}\right)-\bar{\hat{Z}}\left(x_{i}\right)\right]\cdot \left[Z\left(x_{i}\right)-\bar{Z}\left(x_{i}\right)\right]\right\}}^{2}}{\sum_{i=1}^{n}{\left[\hat{Z}\left(x_{i}\right)-\bar{\hat{Z}}\left(x_{i}\right)\right]}^{2}\cdot \sum_{i=1}^{n}{\left[Z\left(x_{i}\right)-\bar{Z}\left(x_{i}\right)\right]}^{2}}$$

where $\hat{Z}\left(x_{i}\right)$ is the measured wetland SOCc, $Z\left(x_{i}\right)$ is the predicted wetland SOCc, $\bar{\hat{Z}}\left(x_{i}\right)$ is the average measured wetland SOCc, $\bar{Z}\left(x_{i}\right)$ is the average predicted wetland SOCc, and *n* is the number of validation sites. The method with the lowest ME and RMSE and highest *r* values was determined as the optimal approach for predicting wetland SOCc in the LRB.

## 3. Results

### 3.1. Descriptive Statistics of Measured Wetland SOCc

The descriptive statistics of all wetland SOCc samples are presented in Table 2. The values of SOCc decrease as the depth increases; averaged values of SOCc are 11.14 (±5.10) g/kg for 0–30 cm, 8.64 (±3.72) g/kg for 30–60 cm, and 8.03 g/kg (±3.38) for 60–100 cm. The variability of SOCc characterized by coefficients of variation (CV) shows moderate variation and declines with soil depth. Wetland SOCc at all soil depths were positively skewed (skewness ≈ 1) and showed a leptokurtic distribution (kurtosis > 0).

### 3.2. Performance of ANN-OK and Comparison of Different Prediction Methods

The parameters of these RBF networks are shown in Table 3. The structures of these RBF networks are 8-27-1, 8-31-1, and 8-23-1; 8 implies that each training point has 8 environmental variable inputs. The optimal numbers of hidden neurons based on the trial-and-error method with the least RMSE are 27 for 0–30 cm, 31 for 30–60 cm, and 23 for 60–100 cm. The values of the output neuron are the predicted wetland SOCc.

The probability distribution of RBF network residuals at all soil depths is consistent with the normal distribution, which is verified by the Kolmogorov-Smirnov (K-S) test with *p* > 0.05. Hence, the residuals were directly used to count the experimental semivariograms for the OK interpolation. Table 4 shows the best-fit experimental variogram parameters of wetland SOCc residuals at three soil depths. The best-fit variogram models for the residuals at 0–30, 30–60, and 60–100 cm are exponential, Gaussian, and spherical models, respectively. The nugget/sill ratios, which reflect the spatial dependence of wetland SOCc residuals, are 43.95%, 47.62%, and 42.64% at 0–30, 30–60, and 60–100 cm, respectively, indicating moderate spatial dependence structures.

The accuracies of different prediction methods for wetland SOCc prediction are presented in Table 5. The performance of OK in wetland SOCc prediction is the worst according to RMSE and *r* but best according to ME. In particular, the correlation between the measured values and predicted values by OK is very low through *r*. Compared with OK, the ANN has better performance. RMSE and *r* have been greatly improved, but ME has decreased. The accuracy of the ANN may be enhanced by the residual prediction of OK. Thus, the ANN-OK has the best prediction results with lower ME, the lowest RMSE, and the highest *r*. Therefore, considering the performance of these methods at different depths, ANN-OK is the best model for wetland SOCc prediction.

**Figure 4:**
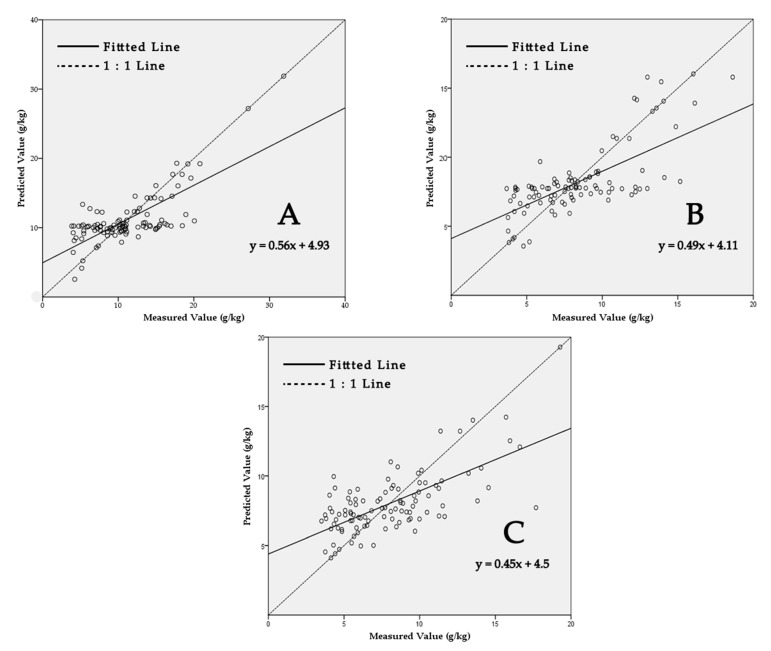
Performance of the hybrid method combining artificial neural network and ordinary kriging (ANN-OK) in predicting wetland soil organic carbon concentration (SOCc) at different depths: (**A**) 0–30, (**B**) 30–60, and (**C**) 60–100 cm.

The 1:1 scatterplot of measured vs. predicted wetland SOCc from the ANN-OK prediction at different depths are shown in Figure 4. ANN-OK explained the spatial dynamics of wetland SOCc to a good extent with large values of *r* being 0.75, 0.71. 0.69 at different depths, respectively. For all depths, wetland SOCc predictions by ANN-OK were credible.

### 3.3. Spatial and Vertical Patterns of Wetland SOCc in the LRB

**Figure 5:**
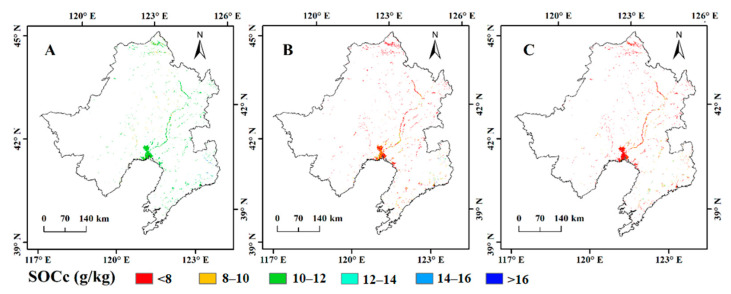
Spatial patterns of the predicted wetland soil organic carbon concentration (SOCc) at different depths: (**A**) 0–30, (**B**) 30–60, and (**C**) 60–100 cm.

The spatial and vertical distributions of wetland SOCc predicted by ANN-OK at different soil depths over the LRB are shown in Figure 5. The distribution pattern of wetland SOCc in the entire study region had great spatial and vertical variability, which tended to decrease from southeast to northwest and from shallow soil depths to deep depths. The ranges of predicted wetland SOCc across the study area at 0–30, 30–60, and 60–100 cm are 2.31–31.52, 2.21–27.93, and 1.11–22.48 g/kg, respectively. Wetland SOCc was concentrated mainly in the topsoil (0–30 cm), and the mean wetland SOCc values at 0–30, 30–60, and 60–100 cm were 10.43 ± 0.38, 7.93 ± 0.25, and 7.61 ± 0.22 g/kg, respectively. The local change in wetland SOCc is apparently observed due to the addition of environmental variables in ANN-OK.

### 3.4. Effects of the Environmental Variables on Wetland SOCc in LRB

Pearson correlation coefficients between wetland SOCc and environmental variables and among various environmental variables at the sampling sites in the LRB are listed in Table 6. Wetland SOCc is significantly linearly correlated with aspect, MAP, and MAH at 0–30 cm; with the NDVI, B5, MAT, MAP, and MAH at 30–60 cm; and with altitude, aspect, MAP, and MAH at 60–100 cm. High collinearity is found among some environmental variables (*r* > 0.6), such as the correlations between NDVI and EVI, band 4 and band 6, and MAH and MAP. The overfitting phenomenon may be caused by the addition of all highly collinear environmental variables in the prediction. By excluding collinear environmental factors and considering the correlation with wetland SOCc, altitude, slope, aspect, NDVI, band 5, band 6, MAT, and MAP were selected as optimal environmental variables to predict wetland SOCc.

**Figure 6:**
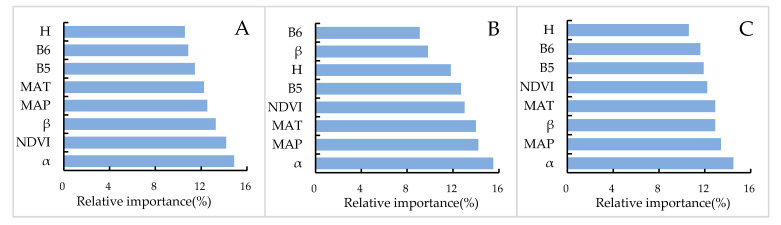
Relative importance of the environmental variables in the prediction of wetland soil organic carbon concentration (SOCc) at different soil depths: (**A**) 0–30 cm, (**B**) 30–60 cm, and (**C**) 60–100 cm.

Figure 6 explains the roles of various environmental factors in wetland SOCc prediction using ANN-OK. At 0–30 cm depth, aspect, which characterizes the land surface orientation conditions, shows the greatest contribution to wetland SOCc prediction, followed by the NDVI, Slope, MAP, and so on. At 30–60 cm, aspect had a larger contribution in predicting wetland SOCc than MAT and MAP. At 60–100 cm, aspect continued to be a primary factor in the prediction of wetland SOCc. MAP and slope were secondary factors, and the contribution of the NDVI further declined. For the prediction of wetland SOCc, with the increase in soil depth, the contribution of NDVI decreased, the relative contribution of climatic factors increased and then decreased, and aspect always performed the most important role at each examined soil depth.

## 4. Discussion

### 4.1. Advantages of ANN-OK in Predicting Wetland SOCc

In this study, wetland SOCc in the LRB was predicted by three approaches such as using only soil samples (i.e., OK), soil samples with environmental variables (i.e., ANN), and a proposed two-step hybrid method combining ANN and OK. The results of ME, RMSE, and *r* indicate that ANN-OK predicted wetland SOCc better, with higher precision and lower uncertainty than other approaches. This result is unanimously consistent with the conclusion of the previous study [19], which mentioned that ANN-integrated residual kriging can provide better prediction accuracy and more detail for soil properties over a large scale than kriging.

In a previous study [35], kriging was considered to have better performance than other pure interpolation methods such as IDW, especially when SOCc data have low sampling density and complex spatial structure. However, the kriging interpolation method performed poorly for the prediction of wetland SOCc in this study. There are two possible reasons for the poor performance of OK. Above all, the strong local variation of wetland SOCc is related to the changes in extrinsic environmental factors, such as vegetation [36], terrain [15], and climate [16] in this study area. Moreover, the spatial correlation of single type soil samples, which may be controlled by the intrinsic factors (i.e., soil properties), is not obvious when they are sparse on a large scale under low sampling density [37]. Hence, these traditional kriging methods are difficult to perfectly describe the spatial and vertical variations of wetland SOCc in the entire study area.

Good performance of ML methods, such as ANN [10], RF [9], boosted regression tree [8], and SVR, in predicting soil parameters were observed from previous studies [7]. In this study, ANN, one of the most popular ML methods, was chosen because of its powerful nonlinear fitting and simple structure [12,13]. Compared to OK which predicts SOCc only on the soil samples, ANN achieved higher accuracy in spatially predicting SOCc, due to its strong performance in gaining more linear or nonlinear relationships between wetland SOCc and environmental variables. ANN does not strongly depend on the sampling density [19], as clarified by the higher accuracy of ANN (Table 5). Despite the better prediction ability they showed, the neglection in spatial autocorrelation indicated that these ML prediction models still can be improved. Compared with ANN, a hybrid model, ANN-OK, achieved good interpretability and predictions of SOCc [38]. Improvement of the ANN-OK can be seen from the low RMSE and the high *r* (Table 5). Therefore, the ANN-OK provides a framework method example for the prediction of other soil parameters at regional scales by means of various environment variables [39]. By employing multisource geospatial data, different soil parameters could be predicted more accurately by such a combination method.

### 4.2. Patterns of Wetland SOCc in the LRB

Although many studies have focused on the spatial variation of wetland SOCc in Northeast China at a local scale [15,16], patterns of wetland SOCc in the entire LRB were not clear due to limited soil investigation and high spatial and vertical variability of soil properties. In the present study, the spatial and vertical patterns of wetland SOCc in the LRB were revealed. The spatial distributions of wetland SOCc in different soil depths showed similar features: higher values were found in the southeast, while lower values were in the northwest. Meanwhile, wetland SOCc decreased with the increase in soil depth and mainly concentrated in the topsoil layer (0–30 cm).

The predicted value of wetland SOCc in the LRB is higher than the average level of China’s wetlands, which is consistent with the previous research result [40]. Different from wetlands in the tropics and subtropics and the arid zone, wetlands in the LRB have high SOCc due to the small decomposition rate caused by low temperature [41,42]. Compared with other wetland distribution regions in Northeast China such as the Greater Khingan Mountains [43] and Western Songnen Plain [44], the values of wetland SOCc in the LRB are relatively low, mostly due to warmer temperature and more decomposition of organic matter in the LRB located in southern Northeast China. The soil microbial activity decreases at low temperatures and high precipitation, which is conducive to stabilizing soil organic carbon storage. Hence, the spatial change of wetland SOCc is similar to spatial distributions of temperatures and precipitation.

For different soil depths, the predicted wetland SOCc values at 0–30 cm have the largest range, and the value range decreases with an increase in depth. This is probably because the spatial heterogeneity and environmental impact on wetland SOCc will decrease with the soil depth increase. In a previous study, the global soil organic carbon was predicted to be mainly concentrated in the topsoil layer (0–30 cm) [45], which is consistent with our observation. This trend demonstrates that the accumulation and emission of soil organic carbon are mainly concentrated in the surface layer of soil [46].

### 4.3. Relationships between Wetland SOCc and Environmental Variables

The physical and chemical properties of soil are affected by environmental conditions [47]. Climate and vegetation significantly impact wetland SOCc distributions, and aspect is the main controlling factor in predicting SOCc. In this study, linear correlations reflected by Pearson correlation coefficients between wetland SOCc and environmental variables were weak (Table 6). This result differs with previous reports [48,49] that SOCc has strong linear correlations with climate and terrain variables, but consistent with another research [19]. These low linear correlations between wetland SOCc and environmental variables probably result from the great changes of topography and climate, as well as the derived change of vegetation across the study area. In summary, the relationships between wetland SOCc and other environmental factors are rarely purely linear [50]. Due to the strong nonlinear fitting ability of machine learning methods, in this study, ANN [29] was used to reveal the complex relationship between environmental factors and wetland SOCc. When we input the same environmental factors, the prediction accuracy decreases with the increase in soil depth, which may imply that the surface organic carbon is more susceptible to environmental factors [43]. Furthermore, surface wetland SOCc can be used as a sensitive indicator of environmental change, which may enhance an ability to detect global climate change.

In this study, aspect contributed the most in predicting wetland SOCc at all soil depths, which is mostly because aspect controls the soil temperature, hydrology condition, and other conditions in a local range to affect the formation of soil organic carbon [51]. However, the value of *r* between aspect and wetland SOCc is small, suggesting they do not have linear relationships. Vegetation debris is an important source of humus formation and organic carbon enrichment [52]. Therefore, NDVI, which reflects vegetation status, has an important contribution in the prediction of wetland SOCc. The relative contribution of NDVI decreased with increased soil depth. This observation is most likely related to limited carbon sequestration due to less vegetation debris in deep soil. Previous studies found that climate conditions significantly contributed to the spatial and vertical distribution of SOCc by affecting respiration of soil microbes, the migration and deposition of soil organic matter, and the growth and rot of vegetation [53]. Generally, wetland SOCc is stable and has no obvious variation even in several years, due to its soil water saturation and anaerobic environment [27]. Therefore, the mean annual meteorological data in ten years were used. For MAP and MAT, the results of this study are consistent with the observation from France [54] that reported precipitation has a greater effect on SOC storage than temperature. Furthermore, with the increase in soil depth, the contribution of climatic factors in predicting wetland SOCc in our study increases first and then decreases.

Soil parameters (such as texture, pH, texture, and so on) are also closely related to the decomposition rate of organic matter and water holding capacity of soil [17], which thus affect the spatial distribution of wetland SOCc. For instance, SOCc is positively correlated to silt content in the Sanjiang Plain [16]. In predicting the spatial pattern of soil parameters by ML, all the environmental input data need to be obtained in geospatial format (polygon or grid). Similar to wetland SOCc, all the other soil parameters also should be spatially interpolated. Therefore, other soil parameters were not considered as input databases in this study. These roles of soil properties can be further explored in examining affecting factors of wetland SOCc.

## 5. Conclusions

In this study, a combination method integrating ANN and OK was used to examine spatial and vertical distributions of wetland SOCc in the LRB. The results showed that ANN-OK was effective in predicting wetland SOCc at a regional scale. Compared with other methods, the prediction accuracy was raised by incorporating ANN and OK with lower RMSE and higher correlation coefficient (*r*). In the LRB, wetland SOCc has obvious spatial and vertical variations. The prediction results showed that wetland SOCc decreased from southeast to northwest, and the high values were mainly concentrated near the ocean, which faces south and has low temperatures, heavy rainfall, and lush vegetation. Wetland SOCc mainly stocked in the topsoil, and the mean wetland SOCc values at soil depths of 0–30, 30–60, and 60–100 cm were 10.43 ± 0.38, 7.93 ± 0.25, and 7.61 ± 0.22 g/kg, respectively, which is larger than the average level of China’s wetland soil but smaller than that over other wetland regions in Northeast China. At the scale of the LRB, terrain aspect contributed the most to predicting wetland SOCc at all soil depths but an insignificant linear correlation between aspect and wetland SOCc was identified. In the future, the impacts of different environmental variables on SOCc could be further analyzed at larger scale. This study provides a framework for the prediction of soil parameters in other regions and it provides a baseline to evaluate the SOCc changes in response to climatic and anthropogenic drivers.

## Figures and Tables

**Table 1:** Environmental variables for wetland soil organic carbon concentration (SOCc) prediction.

Variable Types	Variables	Description
Remote sensing data	B4	Visible-red, 0.64–0.67 μm of the Landsat 8 spectral band
	B5	Near-infrared, 0.85–0.88 μm of the Landsat 8 spectral band
	B6	Short-wave infrared, 1.57–1.65 μm of the Landsat 8 spectral band
	NDVI	Normalized difference vegetation index
	EVI	Enhanced vegetation index
Meteorological data	MAT	Mean annual temperature
	MAP	Mean annual precipitation
	MAH	Mean annual relative humidity
Terrain data	H	Altitude, height above sea level (m)
	β	Slope, the gradient of slope
	α	Aspect, the direction of maximum rate of change

**Table 2:** Descriptive statistics of wetland soil organic carbon concentration (SOCc).

Soil Depth (cm)	Min (g/kg)	Max (g/kg)	Mean (g/kg)	SD (g/kg)	CV (%)	Skewness	Kurtosis
0–30	3.86	31.87	11.14	5.10	45.76	1.03	1.99
30–60	3.67	20.97	8.64	3.72	43.05	1.04	1.17
60–100	3.51	19.28	8.03	3.38	42.10	1.03	0.91

Note: SD: standard deviation. CV: coefficients of variation.

**Table 3:** Parameters of the optimal training ANN model.

Predicted Value	Structure	Hidden Layer Function	Output Layer Function	RMSE
SOCc (0–30 cm)	8-27-1	Gaussian	linear	4.38
SOCc (30–60 cm)	8-31-1	Gaussian	linear	3.09
SOCc (60–100 cm)	8-23-1	Gaussian	linear	2.87

**Table 4:** Parameters for the isotropic semivariogram models for wetland SOCc residuals.

SOCc Residuals	Model	Range (km)	Nugget	Sill	Nugget/Sill (%)
0–30 cm	Exponential	9.6	2.24	5.11	43.95
30–60 cm	Gaussian	3.29	2.00	4.20	47.62
60–100 cm	Spherical	18.70	5.04	11.82	42.64

**Table 5:** Accuracy assessment indicators for the different methods predicting wetland SOCc (means ± standard deviation).

Interval (cm)	Evaluation Indicator	OK	ANN	ANN-OK
0–30	ME	0.09 ± 0.50	−0.08 ± 0.43	−0.02 ± 0.33
	RMSE	5.00 ± 4.39	4.38 ± 2.75	3.37 ± 1.57
	*r*	0.21 *	0.61 **	0.75 **
30–60	ME	−0.08 ± 0.36	−0.18 ± 0.29	−0.15 ± 0.26
	RMSE	3.62 ± 2.17	3.09 ± 2.01	2.65 ± 1.84
	*r*	0.26 **	0.58 **	0.71 **
60–100	ME	−0.02 ± 0.32	−0.13 ± 0.26	−0.01 ± 0.24
	RMSE	3.25 ± 1.77	2.87 ± 1.41	2.47 ± 1.14
	*r*	0.28 **	0.52 **	0.69 **

Note: OK: ordinary kriging; ANN: artificial neural network; ANN-OK: artificial neural network—ordinary kriging; ME: mean error; RMSE: root mean square error; *r*: correlation coefficient; *: *p* < 0. 05; **: *p* < 0.01.

**Table 6:** Correlation coefficients of wetland SOCc and environmental variables.

		SOCc			Altitude	Slope	Aspect	NDVI	EVI	B4	B5	B6	MAT	MAP	MAH
		0–30 cm	30–60 cm	60–100 cm											
SOCc	0–30 cm	1.00													
	30–60 cm	0.64 **	1.00												
	60–100 cm	0.38 **	0.67 **	1.00											
altitude		−0.03	−0.03	−0.16 *	1.00										
slope		−0.05	−0.06	−0.11	0.39 **	1.00									
aspect		0.13 *	0.09	0.16 *	−0.01	0.02 **	1.00								
NDVI		0.10	0.17 *	0.14	0.05 **	0.05 **	0.03 **	1.00							
EVI		0.00	0.08	0.08	0.05 **	0.05 **	0.03 **	0.99 **	1.00						
B4		−0.07	0.00	−0.08	−0.11 **	−0.11 **	−0.01 **	−0.03 **	−0.03 **	1.00					
B5		0.11	0.17 *	−0.01	−0.10 **	0.01	0.00	0.04 **	0.04 **	0.15 **	1.00				
B6		0.05	0.12	−0.10	−0.09 **	−0.01 **	0.00	0.01 **	0.01 **	0.70 **	0.44 **	1.00			
MAT		0.04	0.12 *	0.11	−0.36 **	0.09 **	−0.01	−0.07 **	−0.07 **	0.11 **	0.13 **	0.15 **	1.00		
MAP		0.27 **	0.35 **	0.20 *	−0.21 **	0.30 **	0.00	−0.04 **	−0.04 **	0.03 **	0.09 **	0.10 **	0.54 **	1.00	
MAH		0.20 *	0.33 **	0.30 **	−0.37 **	0.13 **	0.00	−0.10 **	−0.10 **	0.07 **	0.11 **	0.11 **	0.41 **	0.86 **	1.00

Note: * means *p* < 0. 05, ** means *p* < 0.01.
